# Proteins from Modern and Ancient Wheat Cultivars: Impact on Immune Cells of Healthy Individuals and Patients with NCGS

**DOI:** 10.3390/nu14204257

**Published:** 2022-10-12

**Authors:** Walburga Dieterich, Charlotte Schuster, Paulina Gundel, Katharina A. Scherf, Darina Pronin, Sabrina Geisslitz, Andreas Börner, Markus F. Neurath, Yurdagül Zopf

**Affiliations:** 1 Department of Medicine 1, Friedrich-Alexander-Universität Erlangen-Nürnberg, 91054 Erlangen, Germany; 2 Hector-Center for Nutrition, Exercise and Sports, Department of Medicine 1, Friedrich-Alexander-Universität Erlangen-Nürnberg, 91054 Erlangen, Germany; 3 Leibniz-Institute for Food Systems Biology at the Technical University of Munich, 85354 Freising, Germany; 4 Department of Bioactive and Functional Food Chemistry, Institute of Applied Biosciences, Karlsruhe Institute of Technology (KIT), 76131 Karlsruhe, Germany; 5 Leibniz Institute of Plant Genetics and Crop Plant Research (IPK), 06466 Seeland, Germany; 6 Deutsches Zentrum Immuntherapie DZI, 91054 Erlangen, Germany

**Keywords:** wheat, gluten, albumins/globulins, gliadin, immune stimulation

## Abstract

In non-celiac gluten sensitivity (NCGS), the elimination of wheat results in a clear symptom improvement, but gluten has still not been proven as (the sole) trigger. Due to the increase in the prevalence of gluten-related diseases, the breeding of high-performance wheat cultivars is discussed as a trigger. To analyze the immune stimulation and signal pathways, the immune cells of healthy subjects and patients with NCGS were stimulated with gliadins from wheat, and the expression and secretion of interleukin 1ß (IL1ß) and interleukin 6 (IL6) were studied. To determine the impact of wheat breeding, the monocyte cell line THP1 and human immune cells were stimulated with gliadin, glutenin, and albumin/globulin fractions of ancient and modern cereals, and expression of inflammatory molecules was checked. Immune cells of patients with NCGS showed an increased expression of IL1ß and IL6 after stimulation with gliadins compared to immune cells of healthy controls. Gliadins caused a strong activation of P-STAT3 in immune cells of healthy controls, and inhibitors of JAK and NFκB pathways considerably reduced this response. In addition to gliadins, we further showed that glutenins and albumin/globulins from all wheat cultivars from the last century, and especially from einkorn and spelt, also markedly induced the expression of inflammatory genes in THP1 and human immune cells. There was no correlation between enhanced immune stimulation and ancient or modern cultivars. This does not support the hypothesis that modern wheat breeding is responsible for the increase in gluten-related diseases. An altered immune situation is suggested in patients with NCGS.

## 1. Introduction

The prevalence of wheat-related disorders, and in particular celiac disease (CeD) and non-celiac gluten sensitivity (NCGS), has increased in recent years [1,2]. In CeD, the consumption of gluten from wheat, rye, and barley leads to a destructive remodeling of the intestinal mucosa. A hallmark of CeD is gluten-reactive T cells, and in this context especially, the prolamins of wheat (so-called gliadins) are particularly well studied [3].

In contrast, in NCGS, the disease-promoting agent is still unknown. Patients with NCGS experience significant relief of their gastrointestinal and extraintestinal symptoms on a gluten-free diet (GFD), but the evidence as to whether gluten or other components of wheat, such as oligosaccharides, amylase-trypsin inhibitors, or wheat germ agglutinin, are responsible for the development of NCGS is still the subject of numerous studies [1,4]. Patients with NCGS did not show evident mucosal changes except for a slightly increased number of intraepithelial lymphocytes. Interestingly, two-week GFD resulted in a significant reduction in intraepithelial lymphocytes, indicating low-grade inflammation sustained by gluten [5].

Various hypotheses on cereal-associated food intolerances are discussed, particularly the breeding of modern and high-yielding wheat cultivars with disease-promoting properties [6]. Studies by Sofi et al. have shown that consumption of the ancient wheat cultivar *Triticum turgidum*, a progenitor of emmer, over a 6-week period, causes less discomfort and a reduced pro-inflammatory serum cytokine pattern in patients with irritable bowel syndrome than consumption of modern wheat cultivars [7]. In addition, Gianfrani et al. demonstrated that the gliadins of diploid *T. monococcum* differ from the gliadins of hexaploid *T. aestivum* cultivars. Gliadins of *T. monococcum* were more sensitive to enzymatic in vitro digestion and exerted less activation of lymphocytes in ex vivo cultured mucosal biopsies from celiac patients [8]. However, mass spectrometry studies revealed that concentrations of the four most immunoreactive peptides remained almost the same in old and modern wheat cultivars and that the annual climatic conditions (sun, rain, temperature) influenced the protein composition of wheat proteins much more than the respective cultivars [9,10].

It is obvious that the innate immune system is strongly involved in the pathogenesis of NCGS [11]. The incubation of peripheral blood mononuclear cells (PBMCs) of patients with NCGS with wheat proteins resulted in the overexpression of CXCL10 [12,13], which is crucial for the attraction of activated T cells into inflamed tissue. Interestingly, wheat proteins also caused the stimulation of CXCL10 in healthy controls, but serum levels tended to be lower than in NCGS. More ancient wheat cultivars caused a lower overexpression of CXCL10 than modern cultivated cultivars, but the differences did not correlate with the gluten content or specific wheat epitopes [12,13].

The aim of this project was to investigate the impact of gliadins from wheat on the stimulation of immune cells of healthy controls and of patients with NCGS. Since no specific gliadin receptor and no corresponding signaling cascade is known so far, we further analyzed gliadin-induced intracellular signaling. In addition, we analyzed the protein fractions of various ancient and modern wheat cultivars from the last century to investigate the effect of the breeding of wheat cultivars in terms of excessive stimulation of immune cells. Since the diploid and tetraploid wheat varieties are often described as particularly tolerable by patients with irritable bowel syndrome, we also investigated diploid einkorn and tetraploid emmer as well as spelt and rye with regard to the immune response on the human monocyte cell line THP1 and PBMCs from healthy controls.

## 2. Material and Methods

### 2.1. Subjects

Patients with NCGS who clearly responded to a GFD and healthy controls were recruited in the outpatient clinic of the Department of Medicine 1, Universitätsklinikum Erlangen-Nürnberg. The response rate to a GFD was determined according to Catassi et al. [1].

In total, 15 healthy individuals (1 male, 14 females; age 33.5 ± 12.3 years) and 15 patients with suspected NCGS (4 male, 11 females; age 43.5 ± 18.4 years) were included to demonstrate the effect of wheat gliadin on the stimulation of peripheral blood mononuclear cells (PBMCs) before and after a GFD. All participants received professional advice on how to follow a 6-week gluten-free diet, and venous blood was collected before and after the gluten elimination.

In addition, 9 healthy subjects (4 male, 5 females; age 49.4 ± 13.6) and 9 patients with NCGS (1 male, 8 females; age 34.0 ± 11.2) were recruited to perform fluorescence-activated cell sorting analysis of gliadin-induced PBMCs.

Furthermore, 7 healthy individuals (2 male, 5 females; age 32.0 ± 11.1 years) were recruited to determine the effects of various cereal fractions on PBMCs.

Celiac disease and wheat allergy were ruled out by the absence of celiac and wheat-specific antibodies in the serum of all patients and controls. Control subjects were free of gastrointestinal symptoms. The present study includes parts of the clinical trials NCT03268720 (08/15/2017) and NCT04247737 (09/16/2019).

### 2.2. Separation of Cereal Flours into Their Albumin/Globulin, Gliadin, and Glutenin Fractions

Grains were provided by the Leibniz Institute of Plant Genetics and Crop Plant Research in Gatersleben, Germany (IPK). A total of eleven hexaploid wheat cultivars, which were most commonly grown between 1890 and 2010, were cultured in randomized order by IPK and harvested in 2016. 1891–1900: Steigers Leutewitzer Dickkopf, 1901–1910: Ackermanns Brauner Dickkopf, 1911–1920: Stadlers Brauner Dickkopf, 1921–1930: Salzmünder Ella, 1931–1940: Rimpaus Bastard II, 1941–1950: Erbachshofer Braun, 1951–1960: Hadmerslebener Qualitas, 1961–1970: Pilot, 1971–1980: Vuka, 1991–2000: Ritmo, and 2001–2010: Drifter [10]. The grains were milled to wholemeal flour and further separated into their albumin/globulin, gliadin, and glutenin fractions as described earlier [14]. Whole meal flour of other wheat species, e.g., diploid einkorn (Terzino), tetraploid emmer (Ramses), hexaploid spelt (Zollernspelz), and rye, all cultured by IPK in Gatersleben and harvested in 2016, were separated in the same way. The cereal fractions were lyophilized and kept at room temperature until enzymatic digestion. The total content of amylase trypsin inhibitors (ATI) in the flours was determined as described by Geisslitz et al. as follows: wheat cultivar (WC) 1901–1910: 4.85 mg/g; WC 1941–1950: 4.21 mg/g; WC 1951–1960: 4.39 mg/g; WC 1961–1970: 4.15 mg/g; emmer: 4.6 mg/g; spelt: 5.23 mg/g; einkorn: 0.17 mg/g [15].

### 2.3. Enzymatic Digestion of Cereal fractions

The separated wheat fractions were enzymatically digested with pepsin and trypsin. Therefore, 10 mg of each cereal fraction was solubilized in 500 µL 0.1 M hydrochloric acid, pH 1.8, and pepsin (P-7000, Sigma-Aldrich, Steinheim, Germany) was added at 1:100 (*w*/*w*). The digestions were incubated for 4 h at 37 °C; then, the pH value was adjusted to 7.4 with 1 M ammonium bicarbonate buffer, and trypsin (T-9201, Sigma-Aldrich, Steinheim, Germany) was added at 1:100 (*w*/*w*) for a further 4 h at 37 °C. The enzymatic digestions were stopped by addition of 1 µM N-α-Tosyl-L-lysine chloromethyl ketone hydrochloride (Sigma 90182).

Commercially available wheat gliadin (G-3375,Sigma-Aldrich, Steinheim, Germany) was digested with trypsin. A 10 mg amount of gliadin was solubilized in 250 µL 4 M urea for 30 min at 37 °C, and then further diluted with 250 µL of 10 mM ammonium bicarbonate buffer, pH 7.8. Trypsin was added at 1:100 (*w*/*w*), and the suspension was shaken for 4 h at 37 °C. The digestion was stopped by addition of 1 µM N-α-Tosyl-L-lysine chloromethyl ketone hydrochloride.

All digestions were dialyzed (1000 D, Spectra/Por^®^, Serva, Heidelberg, Germany) against 10 mM ammonium bicarbonate buffer, pH 7.8, and afterwards lyophilized. Dried samples were resuspended in phosphate-buffered saline (PBS, Thermofisher Scientific, Dreieich, Germany ) (10 mg/mL). As negative controls, buffers that did not contain any cereal fractions were treated in exactly the same way with enzymes.

### 2.4. Endotoxin Determination

The endotoxin amounts of the cereal digestions were checked with the chromogenic endotoxin quantification kit (PierceTM A39552, Thermofisher Scientific, Dreieich, Germany) according to manufacturer’s instructions and were shown to be <1 endotoxin units per milliliter (EU/mL) in albumin/globulin, and <0.15 EU/mL for glutenin and gliadin culture approaches (1 EU = 0.1 ng endotoxin/mL) (Supplementary Table S1).

### 2.5. Isolation of Peripheral Blood Mononuclear Cells

Venous blood (50 ml) was taken from patients and healthy controls. Peripheral blood mononuclear cells (PBMCs) were isolated by density gradient centrifugation using BioColl separation medium (BS.L6115, Bio&Sell, Feucht, Germany) and Leucosep^®^ tubes (Greiner GN227290, Sigma-Aldrich, Steinheim, Germany) according to manufacturer’s instructions. Isolated PBMCs were washed twice with PBS and used directly for stimulation with cereal fractions. PBMCs that were used for detection of STAT3 protein in Western blot and for flow cytometry were stored in freezing medium (55% RPMI-1640 (Gibco 61870, Thermofisher Scientific, Dreieich, Germany), 35% fetal bovine serum (FBS) (S0615, Biochrom, Berlin, Germany), 10% dimethyl sulfoxide (Honeywell, D5879, VWR International, Darmstadt Germany)) at −150 °C until use. There was no loss in viability after thawing the PBMCs.

### 2.6. Stimulation of THP1 Cells or PBMCs with Cereal Fractions

The human monocyte cell line THP1 was purchased from LGC standards, ATCC TIB-202. The cells were cultured at passage 16 in culture medium (CM: RPMI 1640, 10% FCS, 100 units penicillin/mL, 100 µg streptomycin/mL (Sigma P0781)) in a humidified atmosphere at 37 °C and 5% carbon dioxide (CO_2_). The CM was refreshed every second day, and with increasing density, the cells were diluted at a ratio of 1 to 4.

THP1 cells (2 × 10^6^/ mL) or PBMCs from healthy controls (3 × 10^6^/ mL) were seeded into 24-well culture plates (No 662160, Greiner Bio-One, Frickenhausen, Germany) in 1 mL CM and cultured at 37 °C, 5% CO_2_. Cells were supplemented with 0.5 mg/mL enzymatic digestions of gliadins, glutenins, and albumin/globulins, respectively, and incubated for 24 h. The concentration of albumin/globulins was reduced to 0.1 mg/mL in the PBMC assays because we noticed a negative effect of albumin/globulins on PBMCs at higher concentrations. Afterwards, cells were collected for RNA isolation.

Tryptic digestion of wheat gliadins (Sigma G-3375; 0.5 mg/mL) was used in Western blot experiments, and fluorescence-activated cell sorting (FACS) experiments were conducted to detect differences between controls and patients. Cell culture supernatants were collected and stored at −80 °C until analysis of the secreted cytokines. For FACS analysis, cells were supplemented with a stimulation cocktail (Cell stimulation cocktail 00-4975; eBioscience™, Thermofisher Scientific, Dreieich, Germany) 4 h before cells were collected for staining.

### 2.7. Real-Time Polymerase Chain Reactions (RT-PCR)

RNA extraction was performed with peqGOLD MicroSpin Total RNA Kit (VWR) according to manufacturer’s protocol, and RNA concentrations were determined with a Nanodrop 2000 spectrophotometer (Thermofisher Scientific, Dreieich, Germany). cDNAs were prepared from RNA samples with the iScript cDNA Synthesis Kit (Bio-Rad, Feldkirchen, Germany ). Samples were analyzed in duplicates in RT-PCR with QuantiTect Primer Assay (Qiagen, Hilden, Germany) for gene expression of glyceraldehyde-3-phosphate dehydrogenase (GAPDH; QT00079247, Qiagen, Hilden, Germany), interleukin 1ß (IL1ß; QT00021385, Qiagen, Hilden, Germany), interleukin 6 (IL6; QT00083720, Qiagen, Hilden, Germany), chemokine (C-C motif) ligand 2 (CCL2; QT00212730, Qiagen, Hilden, Germany), and chemokine (C-C motif) ligand 20 (CCL20; QT00012971, Qiagen, Hilden, Germany). Data were calculated using the 2^−ΔΔCt^ method and normalized to GAPDH as a housekeeping gene. The stimulation by cereal peptides was calculated as a ratio compared to cells in pure medium without cereal stimulants.

### 2.8. Cytokine Determination by Enzyme-Linked Immunosorbent Assay (ELISA)

The determination of IL1ß and IL6 protein from cell culture supernatants was performed with commercially available sandwich enzyme-linked immunosorbent assays (human IL-1 beta/IL-1F2 and human IL-6 DuoSet ELISA Kit, R&D systems, Wiesbaden, Germany) according to the manufacturer’s instructions. For all determinations, 100 µL of 1:5 diluted culture supernatant was used, and determination was performed in technical duplicates.

### 2.9. Protein Expression—Western Blot

A total of 3 × 10^6^ freshly isolated or thawed PBMCs were plated in 1 mL CM, supplemented with gliadins (0.5 mg/mL) and cultured at 37 °C and 5% CO_2_ for 24 h. Afterwards, cells were lysed in 300 µL cell lysis buffer (No 9803S, Cell Signaling Frankfurt, Germany) which contained phosphatase inhibitor (PhosSTOP 04906837001, Sigma-Aldrich, Steinheim, Germany) and protease inhibitor cocktail (cOmplete™, Sigma-Aldrich, Steinheim, Germany). The lysates were loaded on 4–20% Tris-Glycine PROTEAN^®^ TGX Stain-Free™ gels (No 456-8095, BioRad Feldkirchen, Germany), and gel electrophoresis was performed. Afterwards, the gels were activated with UV (Gel-Doc™ XR System, BioRad, Feldkirchen, Germany) to visualize fluorescence of proteins. Subsequently, gels were equilibrated in Tris/Glycine buffer which contained 20% (*w*/*v*) methanol, pH 8.3 (BioRad, #1610734). Proteins were plotted on nitrocellulose membranes (Protron 0.2 µm NC, Sigma-Aldrich, Steinheim, Germany), and the fluorescence-activated proteins were recorded (Gel-Doc™ XR System, BioRad, Feldkirchen, Germany). The membranes were washed with Tris-buffered saline (TBS) and blocked overnight (Roti^®^block; A151.4, Roth, Karslruhe, Germany) at 8 °C. Membranes were washed with TBS and incubated with antibodies diluted to 1:500 in TBS/5% bovine serum albumin Fraktion V (No 8076.2, Sigma-Aldrich, Steinheim, Germany) for 3 h at room temperature. 

The following antibodies were used: ß-actin (A5441, Sigma-Aldrich, Steinheim, Germany), p38 MAPK (No 9212, Cell Signaling, Frankfurt, Germany), Phospho-p38 MAPK (No 9211, Cell Signaling, Frankfurt, Germany), STAT1 (No 9175, Cell Signaling, Frankfurt, Germany), Phospho-STAT1 (No 7649, Cell Signaling, Frankfurt, Germany), STAT3 (No 4904, Cell Signaling, Frankfurt, Germany), and Phospho-STAT 3 (Y705) (No 9145, Cell Signaling, Frankfurt, Germany). P-STAT 3 control cell extract (No 9133, Cell Signaling, Frankfurt, Germany) was used to confirm the P-STAT3 protein.

Excess antibodies were removed and membranes were incubated for 1 h at room temperature with peroxidase-labeled goat anti-rabbit IgG or goat anti-mouse IgG, respectively, diluted to 1:5000 in TBS. Membranes were washed and further incubated with 2 mL Immobilon^®^ Forte Western HRP Substrate (Millipore, Merck, Darmstadt, Germany) for 1 min. Fluorescence signals were measured with an Amersham Imager 600 (GE Healthcare, Braunschweig, Germany). Analysis was carried out with Image Lab 6.1 software (BioRad). Normalization was performed by measuring the housekeeping protein band (ß-actin) and adjusting the background.

To check the effect of gliadins on different signaling pathways, the PBMCs were incubated with inhibitors for p38 MAPK (SB202190, Sigma-Aldrich, Steinheim, Germany), for NFκB (Bay 11-7082, Selleckchem, München, Germany), for Smad2 (A8301, Sigma-Aldrich, Steinheim, Germany), and for STAT3 signaling (Tofacitinib, Sigma-Aldrich, Steinheim, Germany). The inhibitors were added to culture medium (10 µM) 1 h before the addition of the tryptic-digested gliadins. The PBMCs were further cultured for 24 h before cells were lysed in cell lysis buffer for Western blot analysis.

### 2.10. Flow Cytometry

Frozen PBMCs from healthy controls and patients with NCGS were thawed and successively washed with PBS and PBS which contained 2% FBS. Next, the cells were incubated with FcR-blocking reagent (No 130-059-901, Miltenyi Biotec, Bergisch Gladbach, Germany). Afterwards, 5 × 10^5^ cells were incubated with anti-CD4 (Horizon™ BV510, BD Biosciences, Heidelberg, Germany) for 30 min at 8 °C in the dark. Then, cells were washed, fixed, and permeabilized with buffer according to manufacturer’s recommendations. Intracellular staining was performed with FoxP3 antibody (APC; 130-093-013; Miltenyi Biotec, Bergisch Gladbach, Germany) for 40 min at 8 °C in the dark. Excess antibodies was removed and cells were resuspended in 350 µL PBS which contained 2% FBS. Acquisition was performed using BD FACSLyric™ and the analysis was performed using FlowJo version 10.6.2. At least 50,000 lymphocytes were gated on size by forward and side scatter. Next, CD4+ cells were selected and FoxP3 expression was analyzed in the CD4+ cell gate. Values have been calculated as percentage of FoxP3+ CD4+ cells / CD4+ cells.

### 2.11. Statistics

Data analysis and graphics were done with GraphPad Prism 8.3.0.

The Wilcoxon signed rank test was used to reveal differences in the gene expression of PBMCs after stimulation with gliadins. The paired *t*-test was assessed to analyze gliadin-induced changes in protein secretion from PBMCs. The Mann–Whitney test was used to compare differences in gene and protein expression of PBMCs induced by gliadins between controls and patients with NCGS.

Differences in STAT3 and P-STAT3 protein expression between controls and patients were analyzed by unpaired *t*-test with Welch’s correction. The one-sample *t*-test was used to compare intragroup-derived data for P-STAT3. The two-tailed paired *t*-test was used to analyze differences in FoxP3+ regulatory T cells after stimulation of PBMCs with gliadins.

One-way ANOVA with multiple-comparison Tukey test was used to analyze differences in gene expression of THP1 cells after stimulation with cereal fractions. The Kruskal–Wallis test with Dunn’s multiple comparisons test was used to analyze differences in gene expression after stimulation of PBMCs of healthy controls with various cereal fractions.

In this study, *p*-values less than 0.05 are considered significant.

## 3. Results

### 3.1. Wheat Gliadins Induce Cytokine Expression in Immune Cells from Healthy Individuals and Patients with NCGS

Oral stimulation with gliadins leads to activation of the native immune system and upregulation of pro- and anti-inflammatory cytokines in patients with NCGS. To determine fundamental differences in inflammatory status, we examined the effect of gliadins on the stimulation of PBMCs from healthy controls and patients with NCGS. Overall, 12 out of 15 patients (80%) with suspected NCGS improved under the GFD with a clear recovery in their three main symptoms, and were therefore classified as patients with NCGS (Supplementary Table S2).

**Figure 1:**
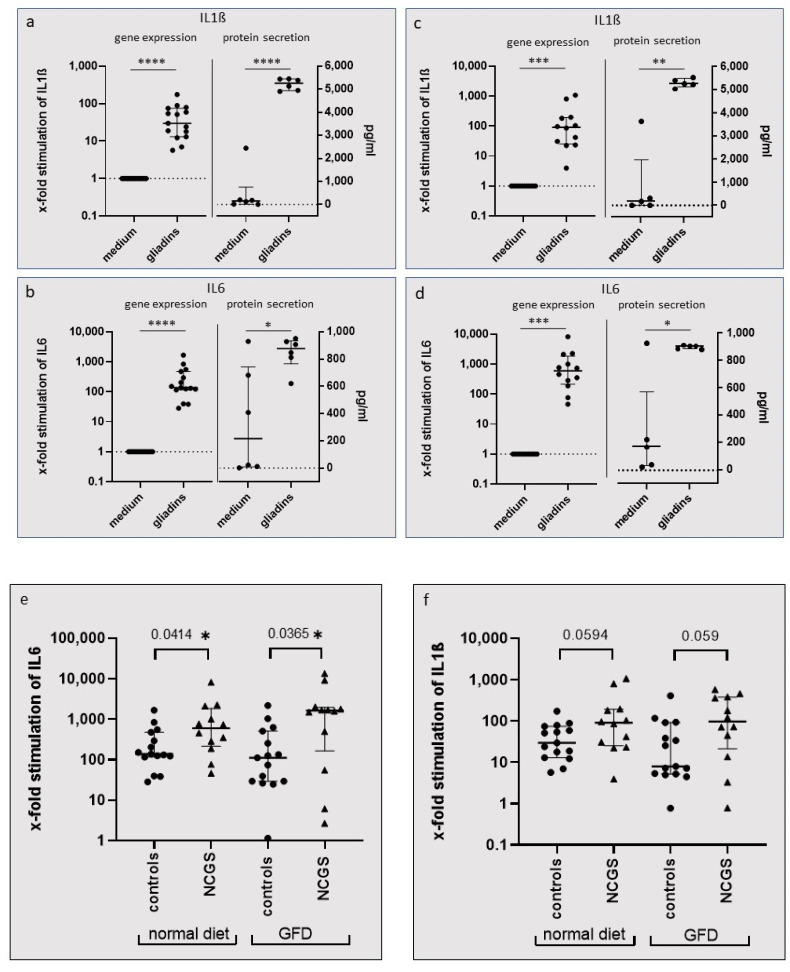
Gene and protein expression of PBMCs after incubation with tryptic-digested gliadins. All results are shown as median with interquartile range. (**a**,**b**) IL1ß and IL6 expression of PBMCs from healthy controls (*n* = 15) and (**c**,**d**) from patients with NCGS (*n* = 12). The Wilcoxon signed rank test revealed significant upregulation of gene expression of PBMCs after stimulation with gliadins. The paired *t*-test revealed significant differences in gliadin-induced protein secretion of PBMCs in healthy controls (*n* = 6) and patients with NCGS (*n* = 5). (**e**,**f**) Gene expression of gliadin stimulated PBMCs under a normal diet and after a 6-week GFD. The Mann–Whitney test was used to detect significant differences in gene expression between NCGS and controls. (**e**) The gliadin-induced expression of IL6 mRNA was significantly higher in the PBMCs of patients with NCGS compared to controls before (with a normal diet) and also after a 6-week GFD. (**f**) Stimulation by gliadins caused a distinct upregulation of IL1ß mRNA in the PBMCs of patients with NCGS compared to controls, although not significantly so. Fold-change gene analysis was carried out with the 2^ΔΔCt^ method, using GAPDH as a housekeeping gene, and was related to values for the culture medium without cereal stimulants as control. * *p* < 0.05; ** *p* < 0.01, *** *p* < 0.005, **** *p* < 0.0001.

PBMCs from controls and patients with NCGS were collected before and after a 6-week GFD and incubated with tryptic-digested wheat gliadins. RT-PCR analysis of PBMC revealed that gliadins caused a significant increase in gene expression of IL1ß and IL6 in the PBMCs of controls and patients with NCGS. Protein determination in cell culture supernatants, which were available from six controls and five patients with NCGS, was performed using ELISA. The data confirmed that gene expression was accompanied by a significantly increased protein secretion of IL1ß and IL6 (Figure 1a–d). Remarkably, gliadins caused a significantly higher gene expression of IL6 in PBMCs from patients with NCGS compared to healthy controls. This was observed both under a normal diet and also after the 6-week GFD (Figure 1e). In addition, IL1ß gene expression was distinctly higher in patients with NCGS before and after the GFD compared to healthy controls, although without showing significance (Figure 1f).

### 3.2. Intracellular Signaling Induced by Gliadins

**Figure 2:**
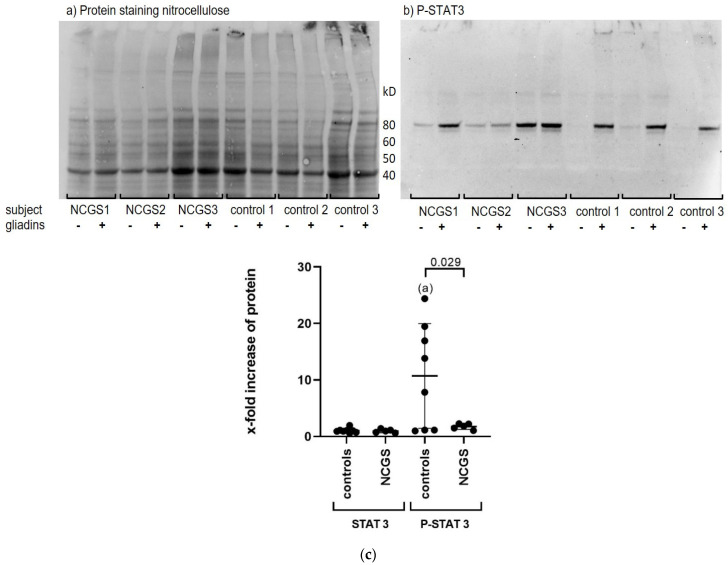
Gliadin-induced phopho-STAT3 protein (P-STAT3) expression in the PBMCs of healthy controls and patients with NCGS. The figure shows representative figures of (**a**) total protein staining (fluorescence) of PBMC lysates separated on 4–12% TGX Stain-Free™ protein gel after transfer to nitrocellulose, and (**b**) Western blot for P-STAT3 in the PBMC of patients with NCGS and healthy controls before and after 24 h stimulation with gliadins. Two biological replicates were performed for *n* = 5 out of 8 healthy controls and *n* = 3 out of 5 patients with NCGS, and one analysis each for the other subjects. Image Lab 6.1 (BioRad) was used to analyze the expression of P-STAT3 protein. Normalization was performed on proteins of 40 kD, i.e., ß-actin (Supplementary Figure S1). (**c**) The figure shows the x-fold enhancement of STAT3 and P-STAT3 protein in the PBMCs from healthy controls and patients with NCGS after stimulation of PBMCs with gliadins compared to a medium without cereal stimulants. Results are shown as median with interquartile range. In the healthy controls, the one-sample *t*-test revealed a significant upregulation of P-STAT3 by gliadins compared to medium control. (**a**) *p* = 0.021. The two-tailed *t*-test with Welch’s correction revealed significant differences in upregulation of P-STAT3 in PBMCs induced by gliadins between healthy controls and NCGS (*p* = 0.029). *p* < 0.5 is considered to be statistically significant.

In order to identify the major signaling pathways in PBMCs induced by stimulation with gliadins, the protein expression of phospho-p38, phospho-STAT1, and phospho-STAT3 was determined by Western blot analysis of PBMC cell lysates. The incubation of cells for 24 h with gliadins caused no upregulation of phospho-p38 and phospho-STAT1.However, gliadin stimulation resulted in a strong upregulation of phospho-STAT3 (P-STAT3) protein in PBMCs from healthy controls (*n* = 8). Remarkably, this upregulation was negligible in patients suffering from NCGS (*n* = 5). This was probably due to the fact that these patients already showed higher levels of P-STAT3 in the basal medium without gliadins, whereas controls have very low basic values (Figure 2).

**Figure 3:**
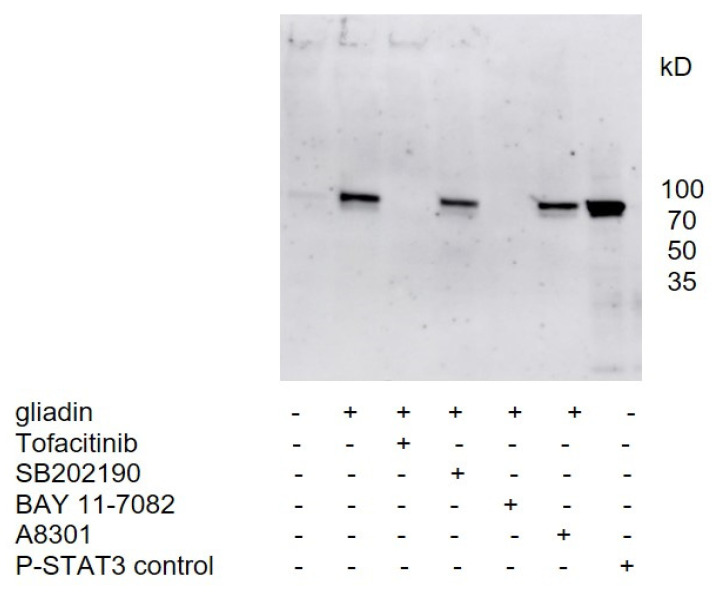
Western blot to detect P-STAT3 upregulation caused by gliadins. Preincubation of PBMCs for 1 h with Tofacitinib citrate. BAY 11-7082 prevented gliadin-induced P-STAT3 expression. No inhibition was noticed with SB202190 and A-8301. The P-STAT3 control protein is shown on the right lane. Experiments were performed twice for *n* = 3 healthy controls, and representative Western blot is shown.

To confirm the activation of STAT3 by gliadins and to study the signaling pathway of gliadin stimulation, PBMCs from healthy controls were used as they showed the strongest effect on P-STAT3 upregulation. Western blot analysis with cell lysates from PBMCs stimulated with gliadins revealed that preincubation of the PBMC with Tofacitinib citrate, a prominent JAK inhibitor with downstream effects on STAT3, prevented the gliadin-induced phosphorylation of STAT3. In addition, the NFκB inhibitor BAY 11-7082, which is also known to attenuate the phosphorylation of activators of STAT3 [16], effectively blocked P-STAT3 upregulation in PBMCs. In contrast, p38 and Alk 4,5,7 inhibitors (SB202190 and A8301, respectively) did not influence gliadin-induced upregulation of P-STAT3 (Figure 3).

### 3.3. Induction of FoxP3 Regulatory T Cells by Gliadins

**Figure 4:**
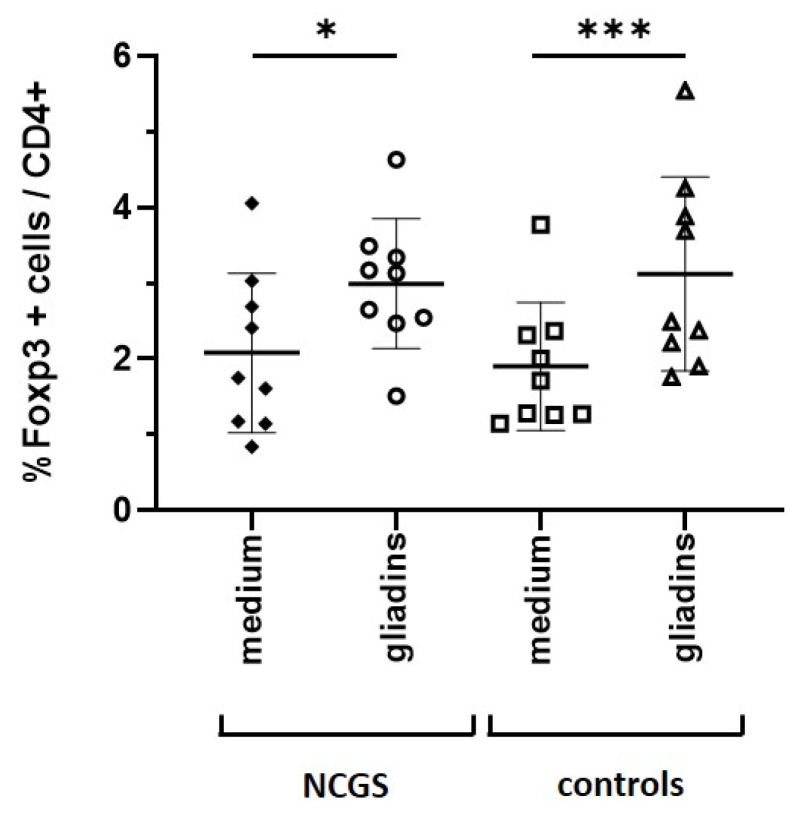
Percentage of FoxP3+ cells in total CD4+ cells. The paired *t*-test revealed significant differences in gliadin-induced increase in FoxP3+ regulatory T cells in patients with NCGS (*n* = 9) and healthy controls (*n* = 9) compared to medium control. * *p* < 0.05, *** *p* < 0.001. Exemplary Fluorescence-activated cell sorting (FACS) panels are shown in Supplementary Figure S2.

It has been shown that STAT3 induces anti-inflammatory responses [17], and the particular role of STAT3 for the expression of forkhead-box-protein 3 (FoxP3) in regulatory T cells has also been demonstrated [18]. Since we noticed that P-STAT3 is strongly upregulated in PBMCs from healthy controls after incubation with gliadins, we further analyzed the influence of gliadins on PBMCs from healthy controls and patients with NCGS in terms of regulatory T cells. PBMCs stimulated with gliadins were stained with CD4 and Foxp3 antibodies and analyzed by FACS. Interestingly, the stimulation of PBMCs with gliadins resulted in a significant enrichment of the percentage of CD4+FoxP3+ regulatory T cells compared to medium control without stimulants (Figure 4). This was noticed in PBMCs derived from healthy controls and individuals suffering from NCGS.

### 3.4. Cereal Fractions Stimulate THP1 Monocyte Cell Lines

Modern wheat breeding is often blamed for the increasing incidence of gluten-related diseases. To determine the effect of cereal proteins and the influence of wheat breeding, eleven of the most commonly cultivated bread wheat cultivars (*Triticum aestivum* subsp. *aestivum*) of the last 100 years were investigated for their immunostimulatory potential. In addition, related wheat species, namely diploid einkorn wheat (*T. monococcum*), tetraploid emmer (*T. turgidum* subsp. *dicoccum*), hexaploid spelt (*T. aestivum* subsp. *spelta*), and rye (*Secale cereale*) were tested.

The human monocyte cell line THP1 was stimulated with the separated and enzymatically digested albumin/globulin, glutenin, and gliadin fractions of all cereals. The endotoxin concentrations were below one endotoxin unit per milliliter (EU/mL) in albumin/globulins and <0.15 EU/mL for glutenins and gliadins in the culture approaches (1EU = 0.1 ng endotoxin/mL; detailed data are available in Supplementary Table S1). Previous experiments showed that a digestion of the cereal fractions with only trypsin yielded similar results to the digestion with pepsin and trypsin (data not shown). Because of their better solubility in culture medium, the double-digested fractions were used for further experiments. The response of THP1 cells to stimulation by cereal fractions was analyzed by the upregulation of inflammatory IL1ß as well as CCL20 and CCL2, which are required for the migration, chemotactic attraction, and maturation of monocytes/macrophages.

**Figure 5:**
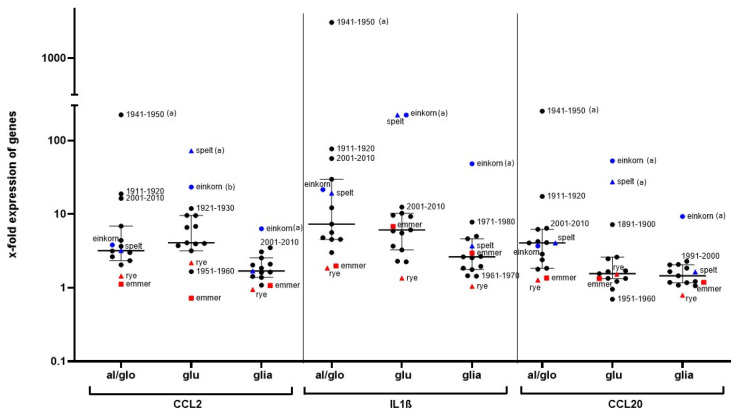
X-fold upregulation of CCL2, IL1ß, and CCL20 in THP1 cells after stimulation with cereal proteins. Gene expression is shown after 24h stimulation of THP1 cells with a peptic-tryptic digestion of albumin/globulin fractions (al/glo), glutenins (glu), or gliadins (glia) (0.5 mg/mL each). Fold-change gene expression was calculated with the 2^−ΔΔCt^ method, using GAPDH as a housekeeping gene, and was related to values for the culture medium without stimulants as a baseline. Data are shown as medians with interquartile range of two independent biological experiments with technical replicates, respectively. One-way ANOVA with multiple Tukey test was used to reveal significant changes in gene expression. (a) Significant differences to all other cereal fractions; (b) significant differences compared to wheat cultivar 1951–1960, rye, spelt, and emmer.

Most of the cereal fractions were able to induce a strong immune response which was characterized by the increased gene expression of CCL2, IL1ß, and CCL20 (Figure 5). The stimulatory potential varied strongly between wheat cultivars from different culture periods and also between cereal species. Interestingly, the salt-soluble albumin/globulin fractions of several wheat cultivars and also the glutenins from einkorn and spelt (displayed in blue color) induced a strong stimulation of THP1 cells. Compared to the albumins/globulins and glutenins, the gliadins possess a rather lower immunostimulatory capacity, with the exception of the gliadins of einkorn. The protein fractions of emmer and rye were consistently characterized by a very low stimulatory capacity (data points labelled in red color). Detailed data are available in Supplementary Table S3.

### 3.5. Cereal Fractions Stimulate Human Immune Cells

The stimulatory effect of the purified cereal fractions was also investigated for human PBMCs. Therefore, bread wheat cultivars that showed low and moderate immunostimulatory potential of THP1 cells (cultivation period 1901–1910, 1951–1960, 1961–1970) and one cultivar with a high stimulatory property (1941–1950) as well as diploid einkorn, tetraploid emmer, hexaploid spelt, and rye were further examined. PBMCs of seven healthy individuals without any history of gut-related diseases and no wheat allergy were isolated and stimulated with the fractions of the selected cereals. Gene expression was determined for inflammation-relevant interleukins IL1ß and IL6 and also CCL20, which are involved in inflammatory processes and chemo-attraction of T and B cells. 

**Figure 6:**
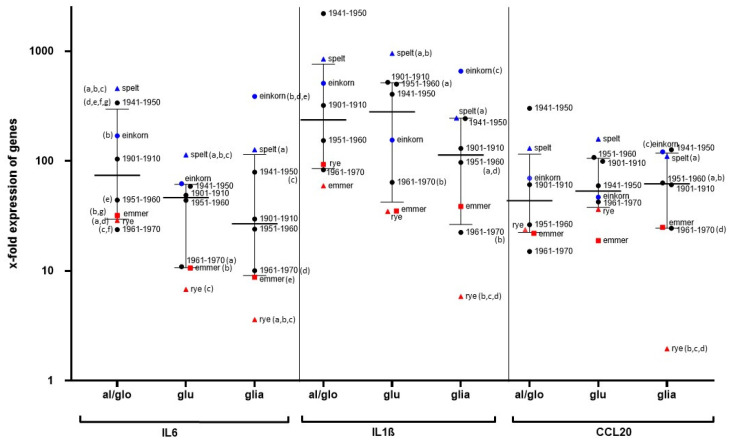
X-fold gene expression of IL6, IL1ß, and CCL20 in the PBMCs of healthy controls (*n* = 7) after stimulation with cereal proteins. Fold-change gene expression was analyzed with the 2^−ΔΔCt^ method, using GAPDH as a housekeeping gene, and was related to values for the culture medium without cereal stimulants as a baseline. Results are shown as medians with interquartile range. The Kruskal–Wallis test with Dunn’s multiple comparisons test was used to analyze differences in gene expression after stimulation of the PBMCs of healthy controls with various cereal fractions. (a,b,c,d) means significant differences between the fractions marked with same letters.

Although individuals’ PBMCs showed considerable variations in gene expression of Il1ß, IL6, and CCL20 after stimulation with cereal fractions, there was high agreement with the data derived from the THP1 cell line. In general, wheat from cultivation period 1961–1970 as well as emmer and rye (red symbols) showed only minor effects on the gene expression of PBMCs from all volunteers. In contrast, incubation with all protein fractions of the wheat cultivar from the period 1941–1950 as well as from einkorn and spelt (blue symbols) led to a strongly increased expression of inflammatory genes (Figure 6). All data are listed in Supplementary Table S4.

## 4. Discussion

In our study, we showed that incubation of PBMCs with wheat gliadins caused a significant increase in gene and protein expression of the inflammatory cytokines IL1ß and IL6. The upregulation was observed under a standard diet and after 6 weeks of GFD in both patients with NCGS and healthy controls, but stimulation was higher in NCGS patients than in the healthy controls.

We could demonstrate that gliadins caused a strong activation of STAT3 in PBMCs. Surprisingly, phosphorylation of STAT3 was more pronounced in the PBMCs of healthy controls, probably because of their very low baseline levels of P-STAT3, whereas PBMCs from patients with NCGS showed higher P-STAT3 levels even before stimulation with gliadins. This is consistent with previous studies, as elevated basal P-STAT3 has already been described in circulating monocytes from active celiac patients compared to patients on a GFD and healthy controls [19], and P-STAT3 expression has previously been shown to be high in the mucosal tissue of patients with active celiac disease [20]. Therefore, it can be speculated that in gluten-sensitive individuals, gluten causes continuous activation of the STAT3 pathway, which affects the regulation of the immune response and immune homeostasis, possibly causing a shift toward proinflammation.

Data from Pallandre et al. indicate a direct role of the STAT3 pathway in the expression of FoxP3, which causes the differentiation of regulatory T cells [18]. Interestingly, we could show that incubation of PBMCs with gliadins resulted in an increased number of FoxP3+ regulatory T cells in both healthy subjects and patients with NCGS. In contrast, Sapone et al. described a reduced expression of FoxP3+ T cell markers in patients with NCGS compared to patients with celiac disease and healthy controls [21]. This discrepancy might be due because we used in vitro stimulated PBMCs and thus recognized a direct differentiation of T cells, whereas Sapone et al. detected gene expression of FoxP3+ from mucosal biopsies and speculated a rather minor recruitment of regulatory T cells into the intestinal mucosa [21]. It is probable that a permanently stressed immune system may favor the loss of oral tolerance, whereas in healthy control subjects, the dietary intake of cereals leads to oral tolerance, characterized by a tightly controlled activation of STAT3 and differentiation of regulatory T cells.

Thus far, no cellular receptor for gliadins is known. We showed that gliadin-induced phosphorylation of STAT3 in the PBMCs of healthy controls could be significantly inhibited by JAK inhibitor (Tofacitinib), and by inhibitors the of classical NFκB pathway (BAY 11-7082), suggesting that signal transduction occurs through these pathways. The involvement of NFκB in gluten signaling has also been suggested since persistent increased activity of NFκB has been observed in patients with CeD on gluten-containing diets, and in vitro cultured intestinal biopsies from patients with CeD showed a marked increase in NFκB activity after stimulation with gliadin peptides [22].

In our study, gene expression of IL1ß and IL6 was upregulated after incubation of PBMCs from healthy controls with the cereal fractions, and of special interest is also the strong stimulation of CCL20 gene expression. CCL20 is part of the first line response to danger-associated molecular patterns and causes a strong chemotactic response and arrest of dendritic cells, effector and memory T cells, and B cells. CCL20 thus possesses an important role in maintaining intestinal homeostatic and inflammatory conditions [23,24,25]. It is also noteworthy that CCL20 is the only chemokine binding to CCR6, which is mainly expressed in memory T cells and mature dendritic cells. Activation of CCR6 causes recruitment of Th17 cells in the small intestine and upregulation of regulatory T cells, making the CCR6/CCL20 axis a crucial element at the cut point between immune suppression or inflammation [23]. CCL20 is involved in the release of IL1ß, which is a strong proinflammatory cytokine and trigger for expression of inflammatory IL6 [26,27]. In turn, stimulation with IL1ß and IL6 induces CCL20 expression via STAT3 signaling, as shown in human periodontal ligament cells, and a continuous activation of NFκB is maintained [28].

The reason for the increasing prevalence of gluten-related diseases is unknown. In particular, it has been discussed whether modern wheat breeding is responsible for the increasing number of patients with gluten-sensitive diseases. In our study, we demonstrated that both ancient and modern wheat cultivars strongly stimulate the monocyte cell line THP1 and human PBMCs in the same way. Although the PBMCs of our subjects responded with marked differences to cereal stimulation, cultivars with generally lower potential (e.g., emmer, rye, Pilot (1961—1970)) and cultivars with strong immune stimulation (spelt, einkorn, Erbachshofer Braun (1941–1950)) could be distinguished. Pronin et al. previously showed that the immunoreactive proteins of the gliadin fraction have not changed in wheat cultivars over the last 100 years [9], and the cultivars examined in our study only showed minor differences in terms of protein composition [10]. Since the ancient wheat cultivar from the cultivation period 1941–1950 elicited the strongest immune response, whereas the more modern cultivars from 1961–1970 caused rather weak immune stimulation, this does not support the hypothesis that wheat breeding is responsible for increased immune triggering leading to an increased prevalence of gluten-related diseases [6].

Other wheat compounds (e.g., ATIs) have been postulated as possible triggers in NCGS rather than gluten. The grains used in our study were characterized by a uniform content of total ATI, as consistent with the literature [15]. Since einkorn had a very low ATI content but strongly stimulated our immune cells, ATI contaminations can be excluded for these stimulatory effects. In addition, a significant effect on the stimulation of PBMCs due to contamination with endotoxin can be excluded, as our glutenin and gliadin fractions of einkorn and spelt had very low endotoxin levels but caused a strong stimulation of PBMC gene expression.

## 5. Summary and Conclusions

As far as we know, we are the first to show that various wheat cultivars are able to stimulate human PBMCs. There is no correlation of enhanced upregulation of inflammatory cytokines/chemokines between modern or ancient wheat cultivars, which does not support the hypothesis that modern wheat breeding triggers gluten-related diseases. In addition to gliadins, we found that albumin/globulins and glutenins also cause strong stimulation of immune cells, so more attention should be paid to these components in future research of gluten-related diseases. Although our cereal fractions contained very small amounts of ATI, we cannot completely rule out effects of other cereal components on immune stimulation in our experiments.

Gliadins caused phosphorylation of STAT3 in PBMCs, and it can be speculated that constant stimulation of the immune system (e.g., by chronic inflammation or gastrointestinal infection) may alter tightly controlled activation of STAT3 and differentiation of Foxp3+ regulatory T cells. This could promote the loss of oral tolerance to glutens and thus favor gluten-related diseases.

Whether adherence to a prolonged GFD can interrupt this permanent activation and restore oral tolerance in patients with NCGS will be clarified in future studies. In daily clinical practice, many patients with NCGS are able to tolerate small amounts of gluten without developing clinical symptoms after having followed a strict 6–8 week GFD, thus suggesting that temporary gluten elimination could stabilize a disturbed immune response.

## Data Availability

Not applicable.

## Supplementary Materials

The following supporting information can be downloaded at: https://www.mdpi.com/article/10.3390/nu14204257/s1, Figure S1: Example of a Western blot to demonstrate ß-actin as 40kD protein; Figure S2: Exemplary FACS panels for CD4+/Foxp3+ staining of PBMC; Table S1: Endotoxin concentrations in culture medium; Table S2: Weekly score of symptoms according the recommended questionnaire for diagnosis of NCGS; Table S3: Upregulation of chemokine C-C motif chemokine ligand 20 (CCL20), C-C motif chemokine ligand 2 (CCL2), and interleukin 1ß (IL1ß) in THP1 cells after stimulation with cereal fractions; Table S4: Upregulation of interleukin 1ß (IL1ß), interleukin 6 (IL6), and C-C motif chemokine ligand 20 (CCL20) after stimulation of PBMC of healthy individuals with cereal fractions.
