# Supplementary material for: Proteins from Modern and Ancient Wheat Cultivars: Impact on Immune Cells of Healthy Individuals and Patients with NCGS
Source: Nutrients. 2022 Oct 12;14(20):4257. doi: 10.3390/nu14204257 (PMC9611902; doi:10.3390/nu14204257)
Supplement: Supplementary file 1 [file nutrients-14-04257-s001.zip › Supplementary Figure 1.pptx]

## Slide 1
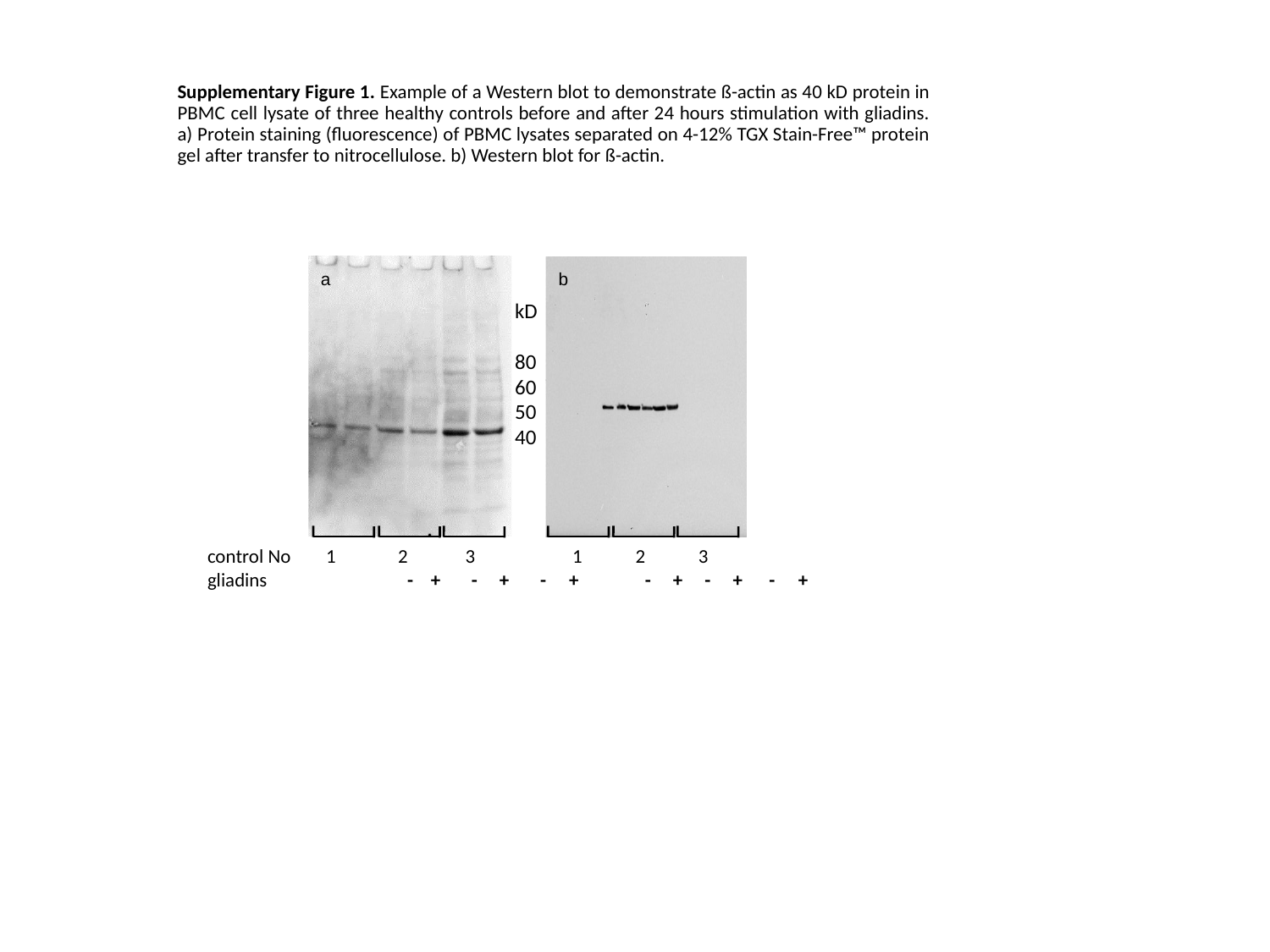

Supplementary Figure 1. Example of a Western blot to demonstrate ß-actin as 40 kD protein in PBMC cell lysate of three healthy controls before and after 24 hours stimulation with gliadins. a) Protein staining (fluorescence) of PBMC lysates separated on 4-12% TGX Stain-Free™ protein gel after transfer to nitrocellulose. b) Western blot for ß-actin.
a
b
kD
80
60
50
40
control No 1 2 3 1 2 3
gliadins	 - + - + - + - + - + - +
