# Supplementary material for: Proteins from Modern and Ancient Wheat Cultivars: Impact on Immune Cells of Healthy Individuals and Patients with NCGS
Source: Nutrients. 2022 Oct 12;14(20):4257. doi: 10.3390/nu14204257 (PMC9611902; doi:10.3390/nu14204257)
Supplement: Supplementary file 1 [file nutrients-14-04257-s001.zip › Supplementary Figure 2.pptx]

## Slide 1
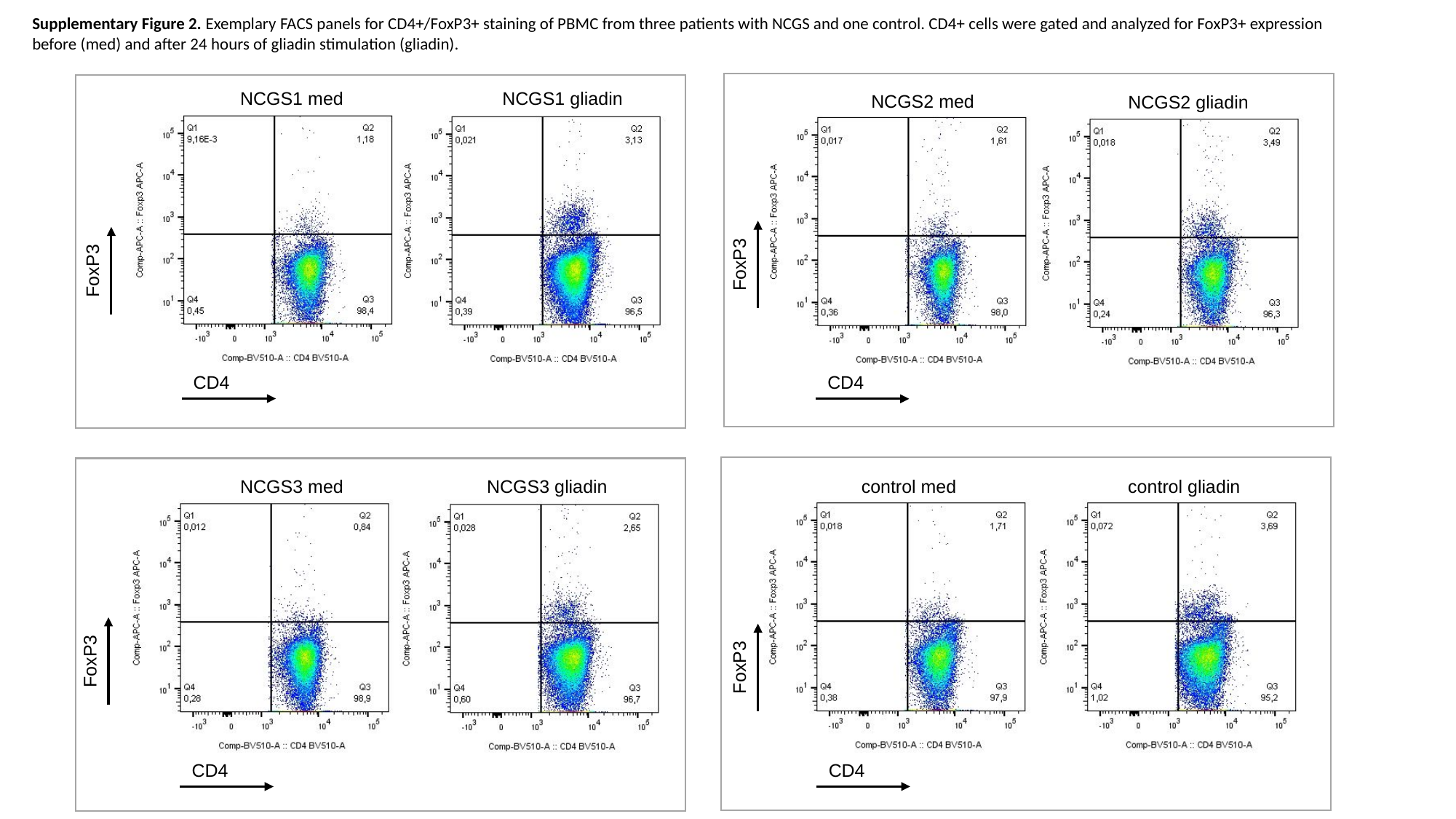

Supplementary Figure 2. Exemplary FACS panels for CD4+/FoxP3+ staining of PBMC from three patients with NCGS and one control. CD4+ cells were gated and analyzed for FoxP3+ expression before (med) and after 24 hours of gliadin stimulation (gliadin).
NCGS1 gliadin
NCGS1 med
NCGS2 med
NCGS2 gliadin
FoxP3
FoxP3
CD4
CD4
NCGS3 gliadin
control med
control gliadin
NCGS3 med
FoxP3
FoxP3
CD4
CD4
