# Supplementary material for: Proteins from Modern and Ancient Wheat Cultivars: Impact on Immune Cells of Healthy Individuals and Patients with NCGS
Source: Nutrients. 2022 Oct 12;14(20):4257. doi: 10.3390/nu14204257 (PMC9611902; doi:10.3390/nu14204257)
Supplement: Supplementary file 1 [file nutrients-14-04257-s001.zip › Supplementary Table 1.pdf]

Supplementary Table S1. Endotoxin concentrations in culture medium

cereal/period	fraction	EU/ml well
1901-1910	al/glo	0.524
1901-1910	glu	0.021
1901-1910	glia	0.045
1941-1950	al/glo	0.941
1941-1950	glu	0.059
1941-1950	glia	0.122
1951-1960	al/glo	0.274
1951-1960	glu	0.033
1951-1960	glia	0.064
1961-1970	al/glo	0.190
1961-1970	glu	0.104
1961-1970	glia	0.048
einkorn	al/glo	0.713
einkorn	glu	0.055
einkorn	glia	0.052
emmer	al/glo	0.329
emmer	glu	0.056
emmer	glia	0.053
spelt	al/glo	0.713
spelt	glu	0.020
spelt	glia	0.054
rye	al/glo	0.455
rye	glu	0.038
rye	secalin	0.129

al/glo = albumin/globulins; glu = glutenin; glia = gliadin; EU = endotoxin units; 1 EU= 0.1 ng endotoxin/ml
