# Supplementary material for: Proteins from Modern and Ancient Wheat Cultivars: Impact on Immune Cells of Healthy Individuals and Patients with NCGS
Source: Nutrients. 2022 Oct 12;14(20):4257. doi: 10.3390/nu14204257 (PMC9611902; doi:10.3390/nu14204257)
Supplement: Supplementary file 1 [file nutrients-14-04257-s001.zip › Supplementary Table 2.pdf]

Supplementary Table S2. Weekly score of symptoms according to the recommended questionnaire for diagnosis of NCGS [2]

patient ID	score three main symptoms		% of improvement	responder[a)]
	**week 0**	**week 6**		
**8**	24	11	54	**x**
**12**	27	14	48	**x**
**20**	23	14	39	**x**
**25**	30	29	3	
**26**	25	13	48	**x**
**27**	24	16	33	**x**
**31**	30	20	33	**x**
**32**	24	19	21	
**33**	29	19	34	**x**
**35**	27	27	0	
**36**	24	12	50	**x**
**37**	21	14	33	**x**
**38**	24	8	67	**x**
**39**	21	13	38	**x**
**41**	19	13	32	**x**

[a)] Patients are defined as responders if they show an improvement of at least >30% in their three main symptoms.
