# Supplementary material for: Proteins from Modern and Ancient Wheat Cultivars: Impact on Immune Cells of Healthy Individuals and Patients with NCGS
Source: Nutrients. 2022 Oct 12;14(20):4257. doi: 10.3390/nu14204257 (PMC9611902; doi:10.3390/nu14204257)
Supplement: Supplementary file 1 [file nutrients-14-04257-s001.zip › Supplementary Table 3.pdf]

Supplementary Table S3. Upregulation of chemokine C-C motif chemokine ligand 20 (CCL20), C-C motif chemokine ligand 2 (CCL2), and interleukin 1ß (IL1ß) in THP1 cells after stimulation with cereal fractions

cereal/ period	CCL20			CCL2			IL1ß		
	al/glo	glu	glia	al/glo	glu	glia	al/glo	glu	glia
1891-1900	4.11 ± 0.48	7.21 ± 2.64	2.05 ± 0.63	3.16 ± 0.80	9.59 ± 2.98	2.09 ± 0.27	4.52 ± 2.31	9.30 ± 1.11	2.63 ± 0.27
1901-1910	1.84 ± 0.13	1.34 ± 0.04	1.06 ± 0.01	2.05 ± 0.30	3.74 ± 0.25	1.38 ± 0.31	5.62 ± 3.25	6.11 ± 2.97	2.55 ± 1.34
1911-1920	17.45 ± 0.28	1.56 ± 0.18	1.46 ± 0.51	18.85 ± 2.76	4.08 ± 1.68	1.64 ± 0.40	76.88 ± 12.03	3.68 ± 2.01	1.46 ± 0.28
1921-1930	2.40 ± 0.59	1.66 ± 0.13	2.07 ± 0.42	3.01 ± 0.43	11.94 ± 6.99	1.85 ± 0.28	3.01 ± 0.96	2.29 ± 0.03	1.85 ± 0.93
1931-1940	4.26 ± 0.07	2.61 ± 0.01	1.22 ± 0.16	3.65 ± 0.23	6.83 ± 1.53	1.42 ± 0.05	7.33 ± 1.65	5.90 ± 2.40	1.95 ± 0.08
1941-1950	250.16 ± 132.15	1.22 ± 0.26	1.08 ± 0.00	223.53 ± 25.51	3.95 ± 1.66	0.97 ± 0.00	3235.35 ± 866.51	10.26 ± 1.24	1.77 ± 0.00
1951-1960	1.79 ± 0.10	0.70 ± 0.11	1.18 ± 0.21	2.33 ± 0.63	1.66 ± 0.59	2.04 ± 0.75	4.79 ± 1.79	2.25 ± 0.52	2.66 ± 0.44
1961-1970	4.06 ± 2.69	2.48 ± 1.55	1.17 ± 0.55	2.64 ± 1.22	4.01 ± 1.89	1.61 ± 0.66	4.54 ± 0.65	3.26 ± 1.92	1.44 ± 0.52
1971-1980	2.86 ± 0.12	0.96 ± 0.36	1.85 ± 0.15	4.40 ± 0.42	6.60 ± 1.78	3.07 ± 0.39	12.20 ± 6.96	9.64 ± 6.06	7.81 ± 5.32
1991-2000	6.22 ± 1.87	1.70 ± 0.47	2.29 ± 0.86	6.87 ± 1.89	3.18 ± 0.07	2.54 ± 0.28	29.77 ± 15.25	5.54 ± 1.91	5.00 ± 2.06
2001-2010	6.43 ± 0.39	1.34 ± 0.30	1.66 ± 0.04	16.34 ± 8.27	9.62 ± 2.69	3.51 ± 1.29	57.02 ± 21.65	12.48 ± 4.14	4.63 ± 0.16
einkorn	3.70 ± 0.03	52.93 ± 11.91	9.31 ± 3.34	3.82 ± 0.63	23.28 ± 9.54	6.34 ± 1.65	21.52 ± 9.67	221.70 ± 33.61	48.30 ± 7.20
emmer	1.35 ± 0.28	1.34 ± 0.45	1.19 ± 0.10	1.12 ± 0.25	0.72 ± 0.15	1.07 ± 0.12	1.97 ± 0.30	6.83 ± 0.26	2.97 ± 1.32
spelt	4.06 ± 0.94	27.45 ± 6.28	1.64 ± 0.06	3.19 ± 1.15	72.50 ± 4.45	1.69 ± 0.06	19.27 ± 0.69	221.23 ± 45.31	3.71 ± 0.49
rye	1.28 ± 0.12	1.53 ± 0.34	0.79 ± 0.12	1.44 ± 0.17	2.18 ± 0.53	0.94 ± 0.08	1.85 ± 0.38	1.35 ± 0.25	1.05 ± 0.01

Gene expression is shown after 24h stimulation of THP1 cells with a peptic-tryptic digestion of albumin/globulin fractions (al/glo), glutenins (glu) or gliadins (glia) (0.5 mg/ml each). Fold-change analysis was done with $2^{-\Delta\Delta Ct}$ method using GAPDH for housekeeping gene and related to values of medium without stimulants as baseline. Data are mean±SD of two independent experiments with technical replicates, respectively. Cereal fractions that are chosen for further experiments with human peripheral blood mononuclear cells are marked in dark grey.
