# Supplementary material for: Proteins from Modern and Ancient Wheat Cultivars: Impact on Immune Cells of Healthy Individuals and Patients with NCGS
Source: Nutrients. 2022 Oct 12;14(20):4257. doi: 10.3390/nu14204257 (PMC9611902; doi:10.3390/nu14204257)
Supplement: Supplementary file 1 [file nutrients-14-04257-s001.zip › Supplementary Table 4.pdf]

**Supplementary Table S4:** Upregulation of interleukin 1ß (IL1ß), interleukin 6 (IL6), and C-C motif chemokine ligand 20 (CCL20) after stimulation of PBMC of healthy individuals with cereal fractions.

| IL1ß | V1 | V2 | V3 | V4 | V5 | V6 | V7 |
|---|---|---|---|---|---|---|---|
| 1901-1910 al/glo | 223.33±67.18 | 7.66 | 27.34 | 292.94 | 35.06 | 125.30 | 18.30 |
| 1901-1910 glu [a)] | 116.86±30.95 | 9.76 | 46.01 | 54.81 | 53.84 | 43.82 | 16.89 |
| 1901-1910 glia | 49.69±1.51 | 6.33 | 34.37 | 54.51 | 6.15 | 21.60 | 34.85 |
| 1941-1950 al/glo | 413.77±46.56 | 30.41 | 84.10 | 1449.86 | 101.12 | 259.07 | 27.83 |
| 1941-1950 glu | 19.07±1.46 | 60.59 | 68.66 | 118.43 | 58.47 | 63.47 | 23.04 |
| 1941-1950 glia | 97.69±3.03 | 62.36 | 28.32 | 157.63 | 13.77 | 163.24 | 31.24 |
| 1951-1960 al/glo | 114.65±46.07 | 5.19 | 11.24 | 41.94 | 18.66 | 103.73 | 13.54 |
| 1951-1960 glu | 27.02±22.41 | 28.71 | 56.38 | 25.96 | 62.82 | 101.23 | 5.12 |
| 1951-1960 glia | 33.61±19.30 | 22.87 | 29.74 | 31.75 | 5.12 | 23.09 | 21.74 |
| 1961-1970 al/glo | 58.26±26.81 | 4.08 | 8.83 | 55.76 | 8.16 | 25.75 | 5.20 |
| 1961-1970 glu | 6.74±5.35 | 9.74 | 19.50 | 26.24 | 6.17 | 7.01 | 1.07 |
| 1961-1970 glia | 7.48±5.45 | 5.77 | 15.67 | 23.54 | 2.14 | 11.65 | 3.97 |
| einkorn al/glo | 282.10±3.83 | 14.31 | 30.87 | 716.04 | 53.14 | 72.50 | 16.69 |
| einkorn glu | 18.67±10.73 | 39.52 | 78.81 | 209.69 | 16.11 | 48.82 | 23.27 |
| einkorn glia | 209.77±110.72 | 80.21 | 32.50 | 2167.08 | 55.03 | 148.88 | 17.40 |
| emmer al/glo | 18.69±4.73 | 3.94 | 10.62 | 156.36 | 11.41 | 15.48 | 6.52 |
| emmer glu | 2.29±1.16 | 25.70 | 12.00 | 22.94 | 2.97 | 5.92 | 2.46 |
| emmer glia | 3.53±0.30 | 2.33 | 23.67 | 17.00 | 1.56 | 4.72 | 8.36 |
| spelt al/glo | 285.63±39.62 | 20.71 | 41.02 | 2606.61 | 69.02 | 139.28 | 30.92 |
| spelt glu [a)] | 62.44±0.84 | 16.90 | 57.60 | 419.87 | 50.45 | 147.80 | 41.28 |
| spelt glia | 92.25±10.23 | 28.27 | 24.03 | 602.29 | 21.80 | 91.28 | 25.69 |
| rye al/glo | 68.58±39.12 | 2.93 | 10.69 | 54.90 | 8.95 | 47.02 | 8.45 |
| rye glu | 4.03±1.19 | 9.15 | 13.69 | 13.75 | 3.15 | 2.88 | 0.78 |
| rye secalin | 4.16±0.23 | 0.98 | 0.65 | 13.02 | 1.84 | 3.82 | 0.76 |

| IL6 | V1 | V2 | V3 | V4 | V5 | V6 | V7 |
|---|---|---|---|---|---|---|---|
| 1901-1910 al/glo | 402.05±231.14 | 207.10 | 814.65 | 85.67 | 274.09 | 258.35 | 199.72 |
| 1901-1910 glu [a)] | 389.04±316.16 | 129.63 | 1874.79 | 24.01 | 649.86 | 197.89 | 376.41 |
| 1901-1910 glia | 44.69±7.64 | 8.74 | 407.12 | 9.50 | 35.41 | 58.32 | 346.11 |
| 1941-1950 al/glo | 3387.33±1837.06 | 2858.72 | 3693.95 | 2220.46 | 931.74 | 1743.25 | 549.08 |
| 1941-1950 glu | 9.79±1.99 | 160.77 | 2086.38 | 10.33 | 79.25 | 242.06 | 249.29 |
| 1941-1950 glia | 202.32±29.42 | 539.19 | 380.78 | 36.69 | 80.18 | 224.24 | 239.20 |
| 1951-1960 al/glo | 172.12±51.90 | 92.11 | 296.37 | 14.58 | 204.97 | 174.12 | 120.84 |
| 1951-1960 glu | 67.66±57.41 | 226.86 | 2147.91 | 15.58 | 632.11 | 326.54 | 86.29 |
| 1951-1960 glia | 50.32±33.75 | 105.18 | 262.69 | 4.37 | 20.71 | 22.96 | 212.79 |
| 1961-1970 al/glo | 65.59±34.20 | 51.61 | 249.27 | 7.87 | 84.74 | 84.45 | 37.93 |
| 1961-1970 glu | 6.33±1.54 | 33.70 | 366.13 | 3.44 | 26.08 | 8.12 | 3.51 |
| 1961-1970 glia | 5.93±2.96 | 5.07 | 89.28 | 1.75 | 19.56 | 14.41 | 20.62 |
| einkorn al/glo | 885.10±285.45 | 702.35 | 818.93 | 143.65 | 404.88 | 334.00 | 273.53 |
| einkorn glu | 5.78±0.47 | 29.49 | 880.34 | 1.52 | 63.58 | 44.21 | 60.99 |
| einkorn glia | 1024.68±358.40 | 827.74 | 605.21 | 547.06 | 615.02 | 668.67 | 308.69 |
| emmer al/glo | 10.50±4.20 | 35.07 | 222.95 | 5.46 | 41.31 | 39.96 | 60.29 |
| emmer glu | 4.97±4.10 | 66.00 | 134.09 | 4.75 | 2.58 | 18.37 | 14.45 |
| emmer glia | 5.54±2.17 | 4.83 | 143.52 | 1.86 | 1.68 | 13.43 | 98.70 |
| spelt al/glo | 1196.47±486.30 | 1034.96 | 1320.54 | 552.40 | 585.02 | 734.16 | 492.31 |
| spelt glu [a)] | 266.06±148.85 | 523.94 | 2500.53 | 79.74 | 556.55 | 1591.50 | 1148.95 |
| spelt glia | 133.47±43.69 | 130.06 | 427.38 | 59.81 | 179.44 | 352.68 | 440.73 |
| rye al/glo | 76.25±47.87 | 22.59 | 302.84 | 8.78 | 70.17 | 97.28 | 73.32 |
| rye glu | 6.78±4.65 | 21.92 | 194.22 | 3.85 | 8.45 | 5.24 | 3.18 |
| rye secalin | 2.43±0.74 | 1.24 | 5.30 | 4.50 | 2.92 | 20.81 | 3.71 |

| CCL20 | V1 | V2 | V3 | V4 | V5 | V6 | V7 |
|---|---|---|---|---|---|---|---|
| 1901-1910 al/glo | 73.34±18.89 | 44.62 | 223.45 | 26.89 | 17.32 | 29.24 | 11.14 |
| 1901-1910 glu [a)] | 96.26±62.08 | 34.41 | 502.99 | 4.87 | 27.12 | 20.84 | 9.35 |
| 1901-1910 glia | 16.04±1.98 | 7.34 | 348.24 | 6.95 | 4.49 | 15.82 | 26.23 |
| 1941-1950 al/glo | 576.11±407.72 | 339.67 | 558.62 | 318.30 | 53.31 | 245.02 | 18.22 |
| 1941-1950 glu | 2.31±1.04 | 37.67 | 331.50 | 5.78 | 13.40 | 13.93 | 11.74 |
| 1941-1950 glia | 29.72±8.74 | 353.18 | 362.20 | 20.58 | 9.27 | 90.49 | 21.10 |
| 1951-1960 al/glo | 39.54±3.99 | 15.24 | 89.04 | 4.78 | 10.62 | 20.79 | 3.78 |
| 1951-1960 glu | 6.76±3.63 | 76.86 | 580.71 | 5.64 | 28.61 | 51.37 | 4.51 |
| 1951-1960 glia | 26.29±22.91 | 64.65 | 301.83 | 4.91 | 4.38 | 17.17 | 23.14 |
| 1961-1970 al/glo | 19.69±4.59 | 7.27 | 54.30 | 3.15 | 5.85 | 12.26 | 2.56 |
| 1961-1970 glu | 3.09±2.48 | 26.97 | 257.46 | 2.13 | 2.01 | 4.06 | 1.17 |
| 1961-1970 glia | 5.91±3.21 | 15.12 | 137.72 | 1.70 | 2.21 | 3.10 | 5.27 |
| einkorn al/glo | 101.54±38.79 | 105.73 | 176.45 | 26.85 | 30.49 | 37.22 | 9.83 |
| einkorn glu | 1.28±0.41 | 22.19 | 280.54 | 0.88 | 7.20 | 8.01 | 7.94 |
| einkorn glia | 135.98±12.34 | 271.57 | 271.32 | 57.51 | 29.78 | 66.47 | 14.62 |
| emmer al/glo | 6.73±4.62 | 6.16 | 109.69 | 1.70 | 8.78 | 10.40 | 10.20 |
| emmer glu | 0.79±0.39 | 30.60 | 84.67 | 0.23 | 1.44 | 9.19 | 4.82 |
| emmer glia | 3.17±1.52 | 9.12 | 127.15 | 1.43 | 2.89 | 8.34 | 22.24 |
| spelt al/glo | 177.07±103.89 | 114.14 | 328.74 | 108.00 | 39.09 | 117.09 | 29.09 |
| spelt glu [a)] | 58.45±14.60 | 75.10 | 817.62 | 15.22 | 28.45 | 85.42 | 28.85 |
| spelt glia | 30.59±5.35 | 61.88 | 419.03 | 13.15 | 28.43 | 161.62 | 56.49 |
| rye al/glo | 34.54±16.31 | 8.27 | 98.20 | 3.54 | 5.08 | 11.42 | 4.60 |
| rye glu | 2.20±1.56 | 16.49 | 229.73 | 0.87 | 1.63 | 2.23 | 1.28 |
| rye secalin | 1.87±0.56 | 1.46 | 2.55 | 1.57 | 1.79 | 3.20 | 1.21 |

Gene expression is shown after stimulation of PBMC of healthy subjects with peptic-tryptic digest (PT) of albumin/globulin fractions (al/glo) (0.1mg/ml), glutenins (glu) (0.5mg/ml) and wheat gliadins (glia) (0.5 mg/ml) or rye secalin (0.5 mg/ml) compared to medium control without stimulants. Fold-change analysis was done with $2^{-\Delta\Delta Ct}$ method using GAPDH for housekeeping gene. a) Stimulation with only 0.25 mg/ml of glutenins. For person V1, two independent biological replicates could be done due to the availability of PBMC. Data are performed in replicates and presented as mean±SD.
